# Feasibility and safety of the discontinuation of systemic immunosuppressive treatment after single-unit cord blood transplantation in adults

**DOI:** 10.1038/s41409-024-02302-6

**Published:** 2024-05-13

**Authors:** Takaaki Konuma, Maki Monna-Oiwa, Seiko Kato, Masamichi Isobe, Yasuhito Nannya, Satoshi Takahashi

**Affiliations:** 1 Department of Hematology/Oncology, The Institute of Medical Science, The University of Tokyo, Tokyo, Japan; 2 Division of Clinical Precision Research Platform, The Institute of Medical Science, The University of Tokyo, Tokyo, Japan

**Keywords:** Bone marrow transplantation, Stem-cell research

## Abstract

We retrospectively evaluated the incidence, factors, and clinical outcomes of the discontinuation of immunosuppressive treatment (IST) after single-unit unrelated cord blood transplantation (CBT) in adults receiving cyclosporine-based graft-versus-host disease (GVHD) prophylaxis at our institute. Among the 309 patients who achieved engraftment, 247 were able to discontinue IST with a median follow-up of 121 months for survivors. The cumulative incidence of the discontinuation of IST was 46.2% at 180 days, 72.8% at 2 years, and 79.3% at 5 years post-CBT. In the multivariate analysis, discontinuation of IST after CBT was significantly associated with the requirement for steroid therapy (hazard ratio [HR]: 0.46; *P* < 0.001) and the recent calendar year of CBT (HR: 1.79; *P* < 0.001). In the conditional landmark analysis at 180 days, discontinuation of IST was not associated with the development of extensive chronic GVHD (HR: 1.00; *P* = 0.989), non-relapse mortality (HR: 0.49; *P* = 0.122), relapse (HR: 1.46; *P* = 0.388), or overall survival (HR: 1.91; *P* = 0.065). Our data showed that successful discontinuation of IST is common after single-unit CBT in adults. Discontinuation of IST did not affect subsequent outcomes, suggesting that discontinuation of IST is both feasible and safe in adults undergoing single-unit CBT.

## Introduction

Allogeneic hematopoietic cell transplantation (HCT) is an established therapeutic modality for intractable hematological disorders. However, graft-versus-host disease (GVHD) remains a significant problem in allogeneic HCT. Therefore, effective GVHD prevention is crucial for the success of allogeneic HCT. Unlike solid organ transplantation, immunosuppressive treatment (IST) used to prevent or treat GVHD can typically be reduced or discontinued in allogeneic HCT recipients, although the optimal timings of such reductions and discontinuations have been unclear [1–3]. In high-risk hematopoietic malignancies, early dose reduction or discontinuation of IST may be used to induce a graft-versus-tumor (GVT) effect, but the efficacy of this approach is not clear [4–9]. Concerns also exist that early reduction or discontinuation of IST may lead to an increase in subsequent chronic GVHD [9–12]. Nevertheless, recent several reports suggest that the early reduction or discontinuation of IST is safe in haploidentical related donor HCT with post-transplant cyclophosphamide [13–15].

When a human leukocyte antigen (HLA)-matched related or unrelated donor is unavailable, cord blood transplantation (CBT) is a well-established therapeutic alternative for adult patients [16–21]. Our previous studies have shown that CBT can produce a more potent graft-versus-leukemia (GVL) effect [22, 23], resulting in a lower post-transplantation relapse rate [24], despite typically lower incidences and severities of chronic GVHD after single-unit CBT compared to conventional adult donor allogeneic HCT [16–19, 21, 25, 26]. Furthermore, T cell immune reconstitution is delayed after CBT compared to conventional adult donor HCT [27, 28]. Given the distinct immunological characteristics following CBT, the optimal timing of dose reduction or discontinuation of IST for GVHD prophylaxis may vary depending on donor type and graft source [1–3], but the clinical management of IST after CBT remains largely unexplored. In this retrospective study, we evaluated the incidence, risk factors, and clinical outcomes associated with the discontinuation of IST following single-unit unrelated CBT in adults using cyclosporine (CSP)-based GVHD prophylaxis at our institute.

## Methods

### Patient selection and transplant procedures

Between August 1998 and September 2023, 336 adult patients underwent single-unit CBT as their initial allogeneic HCT at the Institute of Medical Science, the University of Tokyo. Among them, 16 patients who could not achieve neutrophil engraftment and 11 patients who died without neutrophil engraftment were excluded from this retrospective study. Finally, 309 patients were included in this study. Unrelated cord blood units were supplied by the cord blood bank in Japan. Conditioning regimens, GVHD prophylaxis, and supportive care were determined by the treating physicians according to patient and disease characteristics [29–32]. None of the patients received antithymocyte globulin, alemtuzumab, or rituximab as part of their conditioning regimen or GVHD prophylaxis. Regardless of the development of GVHD or disease relapse, our institute followed almost all patients who were still alive for medications and examinations for at least five years. This retrospective study was approved by the Institutional Review Board of The Institute of Medical Science, the University of Tokyo (2023-55-1019).

### Immunosuppressive treatment schedule

All patients received CSP as a GVHD prophylaxis for CBT in our institute, with or without a short course of methotrexate (MTX) or mycophenolate mofetil (MMF). Patients younger than 59 years and those without comorbidity received CSP and MTX, while patients older than 60 years or those with comorbidity received CSP and MMF [29, 30, 32]. CSP was administered intravenously every day, starting on day −1 at a dose of 3 mg/kg/day. MTX was administered intravenously at 15 mg/m^2^ on days +1, followed by 10 mg/m^2^ on days +3 and +6. MMF was administered orally at a dose of 30 mg/kg/day from day 0 to day +27. We tapered CSP beginning between weeks +4 and +8, withdrawing 10–20% of the starting dose per week, if there was no evidence of active GVHD or mixed chimerism. Patients received oral CSP at a dose of 1:2–2.5, based on their last intravenous dose, once they could tolerate it. Physicians could freely modify the CSP dose for patients experiencing acute GVHD, CSP toxicity, or disease relapse risk. For patients who developed grade II or higher acute GVHD except for the involvement of skin alone, nephrotoxicity, or neurotoxicity mainly due to CSP, CSP was discontinued and switched to steroid therapy. Finally, in the absence of GVHD, we could discontinue CSP or steroids between 3 and 12 months after CBT.

### Definitions

The steroid therapy was defined as systemic administration equivalent to 0.5 mg/kg/day or more prednisolone after CBT. Discontinuation of IST was defined as the first data of discontinuation of all systemic administration of immunosuppressive drugs, such as calcineurin inhibitors, and steroids. The degree of HLA matching was determined based on antigenic levels for HLA-A, HLA-B, and HLA-DR loci in the graft-versus-host direction. The HCT-Specific Comorbidity Index (HCT-CI) [33] and refined Disease Risk Index (DRI) [34] were classified according to published criteria. The diagnosis of acute and chronic GVHD was based on previously established standard criteria [35, 36]. Relapse was defined as the hematological recurrence of the underlying hematological disease. Non-relapse mortality (NRM) was defined as death without hematological recurrence of the underlying hematological disease. Disease-free survival (DFS) was defined as the time from the date of CBT until death, relapse, or survival. Overall survival (OS) was defined as the time from the date of CBT until death or survival.

### Statistical analysis

The probability of the discontinuation of IST, extensive chronic GVHD, NRM, and relapse was calculated using cumulative incidence curves, taking into account competing risks, and Gray’s test was used for group comparisons. The competing event for discontinuation of IST was relapse, death, or late graft failure before the event. Death before the event was considered the competing event for extensive chronic GVHD. Regarding NRM, relapse was a competing event, and vice versa. The probability of DFS and OS was calculated using the Kaplan–Meier method, with group comparisons performed using the log-rank test.

Factors influencing the discontinuation of IST were evaluated using the Fine and Gray model in the univariate and multivariate analyses, using the following factors: the requirement for steroid therapy (no requirement vs. requirement), age (<45 vs. ≥45 years), HCT-CI (0–2 vs. ≥ 3), disease type (myeloid vs. other than myeloid), refined DRI (low/intermediate vs. high/very high), cryopreserved cord blood total nucleated cell (TNC) dose (<2.5 × 10^7^/kg vs. ≥2.5 × 10^7^/kg), number of HLA disparities (0, 1 vs. 2), sex incompatibility (female donor to male recipient vs. others), ABO incompatibility (match, minor mismatch vs. major, bidirectional mismatch), and year of CBT (1998–2009 vs. 2010–2023), with the requirement for steroid therapy treated as a time-dependent covariate.

To clarify the impact of the discontinuation of IST on extensive chronic GVHD, NRM, relapse, DFS, and OS following single-unit CBT, multivariate analysis was performed using the Fine and Gray model for extensive chronic GVHD, NRM, and relapse, and a Cox proportional hazards model for DFS and OS. In these models, the discontinuation of IST and the requirement for steroid therapy were treated as time-varying covariates. The following factors associated with the impact of the discontinuation of IST on extensive chronic GVHD, NRM, relapse, DFS, and OS were considered in the multivariate analysis: the situation of IST (discontinuation of IST vs. continuation of IST), requirement for steroid therapy (no requirement vs. requirement), age (<45 vs. ≥45 years), HCT-CI (0–2 vs. ≥ 3), disease type (myeloid vs. other than myeloid), refined DRI (low/intermediate vs. high/very high), cryopreserved cord blood TNC dose (<2.5 × 10^7^/kg vs. ≥2.5 × 10^7^/kg), number of HLA disparities (0, 1 vs. 2), sex incompatibility (female donor to male recipient vs. others), ABO incompatibility (match, minor mismatch vs. major, bidirectional mismatch), and year of CBT (1998–2009 vs. 2010–2023). Age and cord blood TNC were categorized based on the approximate median value. The impact of the discontinuation of IST on transplant outcomes was evaluated using a landmark analysis at day 180 after CBT, corresponding to the date by which around 50% of patients had discontinued IST.

All *P*-values were two-sided, and the data were analyzed using EZR version 1.61 (Saitama Medical Center, Jichi Medical University, Saitama, Japan) [37], a graphical user interface for the R 4.2.3 software program (R Foundation for Statistical Computing, Vienna, Austria). *P*-values < 0.05 were considered to be significant.

## Results

### Patient characteristics and GVHD incidences

Table 1 summarizes the baseline characteristics and CBTs of the 309 patients. At the time of CBT, the median age of recipients was 44 years (interquartile range [IQR]: 33–51 years). Of the patients, 183 (59.2%) were male, and 36 (12.3%) had an HCT-CI ≥ 3. The most common indications for CBT were acute myelogenous leukemia (*n* = 169; 54.7%), followed by acute lymphoblastic leukemia (*n* = 61; 19.7%) and myelodysplastic syndrome (*n* = 37; 12.0%). The majority of conditioning regimens were total body irradiation 12 Gy, cyclophosphamide 120 mg/kg, and high-dose cytarabine 12 g/m^2^ (*n* = 228; 73.8%) [29, 32]. The most common GVHD prophylaxis was CSP and MTX (*n* = 260; 84.1%), followed by CSP and MMF (*n* = 46; 14.9%) and CSP alone (*n* = 3; 1.0%). The median cryopreserved cord blood TNC dose and CD34^+^ cell dose was 2.53 × 10^7^/kg (IQR: 2.15–3.04 × 10^7^/kg) and 0.98 × 10^5^/kg (IQR: 0.72–1.26 × 10^5^/kg), respectively. The number of HLA antigen mismatches at HLA-A, -B, and -DRB1 between recipients and cord blood units was 0 or 1 in 108 (35.0%) patients and 2 in 201 (65.0%) patients. Regarding sex compatibility, 89 (28.8%) patients received a female donor to male recipient transplant (Table 1).

**Table 1:** Patient characteristics.

Characteristic	Value
Number of patients	309
Age at CBT, years, median (IQR)	44 (33–51)
Sex, numbers (%)	
Male	183 (59.2)
Female	126 (40.8)
HCT-CI, numbers (%)	
0–2	256 (87.7)
≥3	36 (12.3)
Disease type, numbers (%)	
AML	169 (54.7)
ALL	61 (19.7)
MDS	37 (12.0)
NHL/ATL	15 (4.9)
CML	13 (4.2)
CMML	4 (1.3)
CAEBV	3 (1.0)
MPN	2 (0.6)
MM	2 (0.6)
SAA	2 (0.6)
Mastocytosis	1 (0.3)
Refined disease risk index, numbers (%)	
Low/Intermediate	163 (54.5)
High/Very high	136 (45.5)
Not available	10
Conditioning regimen, numbers (%)	
TBI 12 Gy+CY + HDCA ± G-CSF	228 (73.8)
TBI 8–12 Gy+others	36 (11.7)
TBI 4 Gy+BU + FLU + HDCA + G-CSF	38 (12.3)
TBI 2–4 Gy+others	7 (2.3)
GVHD prophylaxis, numbers (%)	
CSP with MTX	260 (84.1)
CSP with MMF	46 (14.9)
CSP alone	3 (1.0)
Cryopreserved cord blood TNC dose, x10^7^/kg, median (IQR)	2.53 (2.15–3.04)
Cryopreserved cord blood CD34^+^ cell dose, x10^5^/kg, median (IQR)	0.98 (0.72–1.26)
Cryopreserved cord blood CFU-GM dose, x10^3^/kg, median (IQR)	27.06 (19.92–38.85)
HLA disparities ^a^, numbers (%)	
0, 1	108 (35.0)
2	201 (65.0)
Sex incompatibility, numbers (%)	
Female donor to male recipient	89 (28.8)
Others	220 (71.2)
ABO incompatibility, numbers (%)	
Match	85 (27.5)
Minor mismatch	86 (27.8)
Major mismatch	87 (28.2)
Bidirectional mismatch	51 (16.5)
Year of CBT, numbers (%)	
1998–2009	152 (49.2)
2010–2023	157 (50.8)

*CBT* cord blood transplantation, *IQR* interquartile range, *HCT-CI* hematopoietic cell transplantation specific comorbidity index, *AML* acute myeloid leukemia, *ALL* acute lymphoblastic leukemia, *MDS* myelodysplastic syndrome, *NHL* non-Hodgkin’s lymphoma, *ATL* adult T-cell leukemia, *CML* chronic myelogenous leukemia, *CMML* chronic myelomonocytic leukemia, *CAEBV* chronic active Epstein-Barr virus infection, *MPN* myeloproliferative neoplasm, *MM* multiple myeloma, *SAA* severe aplastic anemia, *TBI* total body irradiation, *CY* cyclophosphamide, *HDCA* high-dose cytarabine, *G-CSF* granulocyte colony-stimulating factor, *BU* busulfan, *FLU* fludarabine, *GVHD* graft-versus-host disease, *CSP* cyclosporine, *MTX* methotrexate, *MMF* mycophenolate mofetil, *TNC* total nucleated cell, *CFU-GM* colony-forming unit granulocyte-macrophage, *HLA* human leukocyte antigen.

^a^HLA disparities between cord blood graft and recipient were defined as a low-resolution for HLA-A, HLA-B, and HLA-DR in the graft-versus-host direction.

In the entire cohort, the cumulative incidence of grade II–IV acute GVHD and grade III–IV acute GVHD at 100 days was 70.9% (95% confidence interval [CI]: 65.5–75.6%) and 13.0% (95% CI: 9.5–17.0%), respectively. Of the 219 patients who developed grade II–IV acute GVHD, 90 (41.0%) required steroid therapy. Of the 40 patients who developed grade III–IV acute GVHD, 38 (95.0%) required steroid therapy.

### Factors and incidences of discontinuation of IST

With a median follow-up of 121 months (IQR: 67–279 months) for survivors, 247 patients discontinued IST, whereas 8 patients continued it. Among the remaining 54 patients, 27 experienced a hematological recurrence of underlying hematological disease, 26 died of NRM, and one experienced late graft failure, all of which are competing events for discontinuation of IST.

**Fig. 1:**
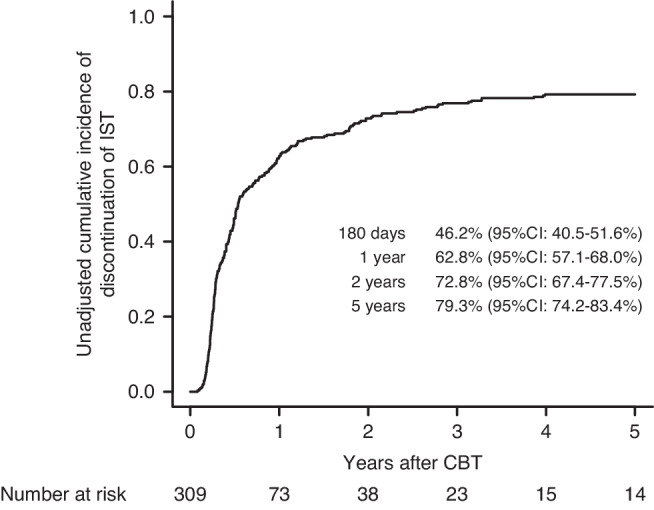
Incidence of immunosuppressive treatment discontinuation after cord blood transplantation. Unadjusted cumulative incidence of immunosuppressive treatment (IST) discontinuation following single-unit cord blood transplantation (CBT).

The cumulative incidence of the discontinuation of IST was 25.9% (95% CI: 21.2–30.9%) at 100 days, 46.2% (95% CI: 40.5–51.6%) at 180 days, 62.8% (95% CI: 57.1–68.0%) at 1 year, 72.8% (95% CI: 67.4–77.5%) at 2 years, and 79.3% (95% CI: 74.2–83.4%) at 5 years post-CBT (Fig. 1).

In the univariate analysis using the Fine and Gray proportional hazards model, the requirement for steroid therapy was significantly associated with a decreased incidence of the discontinuation of IST after CBT (hazard ratio [HR]: 0.44; 95% CI: 0.33–0.58; *P* < 0.001), whereas immunosuppression was discontinued significantly more frequently in transplants performed more recently (HR: 1.46; 95% CI: 1.14–1.89; *P* = 0.003) (Table 2). In the multivariate analysis using the Fine and Gray proportional hazards model, the requirement for steroid therapy (HR: 0.46; 95% CI: 0.34–0.63; *P* < 0.001) and the recent calendar year of CBT (HR: 1.79; 95% CI: 1.35–2.37; *P* < 0.001) remained significant factors for the discontinuation of IST after CBT (Table 2).

**Table 2:** Univariate and multivariate analysis of discontinuation of IST.

	Univariate analysis		Multivariate analysis	
	HR (95% CI)	*P*-value	HR (95% CI)	*P*-value
Requirement for steroid therapy				
No requirement for steroid	Reference		Reference	
Requirement for steroid	0.44 (0.33–0.58)	**<0.001**	0.46 (0.34–0.63)	**<0.001**
Age at CBT				
<45 years	Reference		Reference	
≥45 years	0.92 (0.72–1.18)	0.550	0.78 (0.60–1.03)	0.082
HCT-CI				
0–2	Reference		Reference	
≥3	0.97 (0.65–1.45)	0.890	0.93 (0.59–1.45)	0.767
Disease type				
Myeloid	Reference		Reference	
Other than myeloid	1.21 (0.90–1.63)	0.200	1.16 (0.83–1.62)	0.373
Refined DRI				
Low/Intermediate	Reference		Reference	
High/Very high	0.81 (0.63–1.06)	0.130	0.91 (0.68–1.22)	0.570
Cord blood TNC				
<2.5 × 10^7^/kg	Reference		Reference	
≥2.5 × 10^7^/kg	1.20 (0.94–1.54)	0.140	1.14 (0.87–1.50)	0.319
HLA disparities				
0, 1	Reference		Reference	
2	1.19 (0.91–1.56)	0.190	1.10 (0.83–1.62)	0.373
Sex incompatibility				
Female donor to male recipient	Reference		Reference	
Others	1.09 (0.83–1.44)	0.520	1.06 (0.79–1.43)	0.677
ABO incompatibility				
Match, minor mismatch	Reference		Reference	
Major, bidirectional mismatch	0.90 (0.70–1.16)	0.430	0.86 (0.66–1.13)	0.294
Year of CBT				
1998–2009	Reference		Reference	
2010–2023	1.46 (1.14–1.89)	**0.003**	1.79 (1.35–2.37)	**<0.001**

The *P* values in bold are statistically significant (<0.05).

*CBT* cord blood transplantation, *HCT-CI* hematopoietic cell transplantation specific comorbidity index, *DRI* disease risk index, *TNC* total nucleated cell, *HLA* human leukocyte antigen, *HR* hazard ratio, *CI* confidence interval.

We also evaluated the effects of the development of acute GVHD on the discontinuation of IST. In the univariate analysis using the Fine and Gray proportional hazards model, in which the development of acute GVHD was treated as a time-dependent covariate, we found that developed grade III-IV acute GVHD was significantly associated with a decreased incidence of the discontinuation of IST after CBT (HR: 0.36; 95% CI: 0.23–0.58; *P* < 0.001), but not developed grade II-IV acute GVHD (HR: 1.01; 95% CI: 0.77–1.33; *P* = 0.892).

### Impact of discontinuation of IST on extensive chronic GVHD

**Fig. 2:**
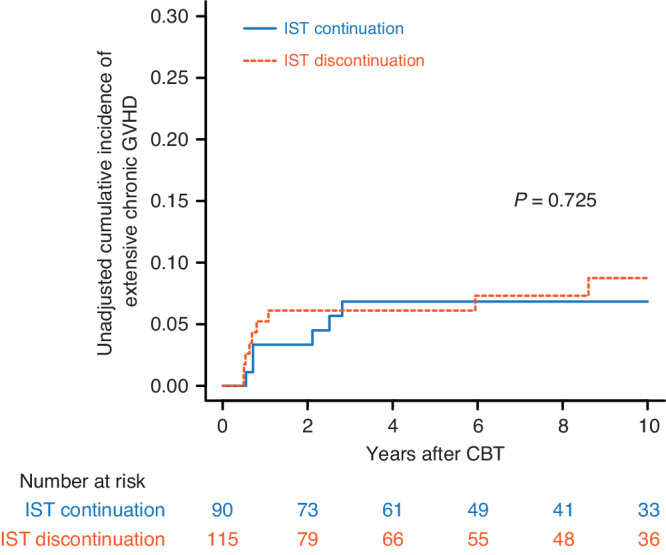
Extensive chronic graft-versus-host disease after cord blood transplantation according to immunosuppressive treatment discontinuation. Unadjusted cumulative incidence of extensive chronic graft-versus-host disease (GVHD) following single-unit CBT, according to IST discontinuation, plotted with a conditional landmark analysis at 180 days after CBT.

In the conditional landmark analysis at 180 days, the cumulative incidence of extensive chronic GVHD at 5 years was 6.8% (95% CI: 2.8–13.4%) for patients who continued IST and 6.1% (95% CI: 2.7–11.5%) for patients who discontinued IST (*P* = 0.725 by Gray’s test) (Fig. 2). In the multivariate analysis, when continuation of IST was used as the reference group, discontinuation of IST was not associated with the development of extensive chronic GVHD (HR: 1.00; 95% CI: 0.27–3.72; *P* = 0.989) (Table 3).

**Table 3:** Multivariate analysis of extensive chronic GVHD, NRM, relapse, treatment failure (1-DFS), and overall mortality (1-OS).

	Extensive chronic GVHD		NRM		Relapse		Treatment failure (1-DFS)		Overall mortality (1-OS)	
	HR (95% CI)	*P*-value	HR (95% CI)	*P*-value	HR (95% CI)	*P*-value	HR (95% CI)	*P*-value	HR (95% CI)	*P*-value
Situation of ITS										
IST continuation	Reference		Reference		Reference		Reference		Reference	
IST discontinuation	1.00 (0.27–3.72)	0.989	0.49 (0.20–1.21)	0.122	1.46 (0.61–3.46)	0.388	0.87 (0.47–1.61)	0.676	1.91 (0.95–3.82)	0.065
Requirement for steroid therapy										
No requirement for steroid	Reference		Reference		Reference		Reference		Reference	
Requirement for steroid	0.21 (0.02–2.04)	0.182	1.88 (0.92–3.84)	0.081	1.05 (0.51–2.17)	0.887	1.44 (0.86–2.39)	0.156	1.67 (1.02–2.72)	**0.037**
Age at CBT										
<45 years	Reference		Reference		Reference		Reference		Reference	
≥45 years	2.11 (0.47–9.33)	0.324	4.52 (2.00–10.21)	**<0.001**	1.26 (0.67–2.35)	0.467	2.19 (1.33–3.60)	**0.002**	2.28 (1.40–3.70)	**<0.001**
HCT-CI										
0–2	Reference		Reference		Reference		Reference		Reference	
≥3	5.21 (1.34–20.28)	**0.017**	1.56 (0.60–4.06)	0.354	0.84 (0.32–2.20)	0.729	0.96 (0.48–1.91)	0.910	1.26 (0.66–2.39)	0.476
Disease type										
Myeloid	Reference		Reference		Reference		Reference		Reference	
Other than myeloid	3.26 (1.03–10.34)	**0.044**	1.62 (0.65–4.01)	0.297	1.43 (0.69–2.94)	0.328	1.70 (0.98–2.94)	0.059	1.39 (0.80–2.42)	0.236
Refined DRI										
Low/Intermediate	Reference		Reference		Reference		Reference		Reference	
High/Very high	0.65 (0.15–2.74)	0.566	1.58 (0.72–3.47)	0.253	3.35 (1.79–6.27)	**<0.001**	2.88 (1.79–4.65)	**<0.001**	3.11 (1.92–5.04)	**<0.001**
Cord blood TNC										
<2.5 × 10^7^/kg	Reference		Reference		Reference		Reference		Reference	
≥2.5 × 10^7^/kg	1.67 (0.51–5.43)	0.389	0.81 (0.38–1.71)	0.587	1.06 (0.58–1.94)	0.843	0.96 (0.61–1.53)	0.889	0.84 (0.53–1.31)	0.451
HLA disparities										
0, 1	Reference		Reference		Reference		Reference		Reference	
2	1.95 (0.31–12.26)	0.476	1.32 (0.62–2.82)	0.461	0.82 (0.44–1.53)	0.551	1.05 (0.65–1.70)	0.823	1.32 (0.81–2.16)	0.252
Sex incompatibility										
Female donor to male recipient	Reference		Reference		Reference		Reference		Reference	
Others	0.25 (0.04–1.37)	0.112	0.62 (0.28–1.33)	0.222	0.84 (0.42–1.68)	0.636	0.71 (0.43–1.15)	0.169	0.58 (0.36–0.92)	**0.020**
ABO incompatibility										
Match, minor mismatch	Reference		Reference		Reference		Reference		Reference	
Major, bidirectional mismatch	4.49 (1.45–13.89)	**0.009**	0.99 (0.44–2.19)	0.984	1.18 (0.64–2.15)	0.583	1.17 (0.73–1.88)	0.496	0.95 (0.59–1.52)	0.848
Year of CBT										
1998–2009	Reference		Reference		Reference		Reference		Reference	
2010–2023	0.25 (0.06–1.01)	0.053	0.48 (0.21–1.09)	0.080	1.06 (0.53–2.11)	0.855	0.90 (0.53–1.53)	0.707	0.64 (0.39–1.06)	0.085

The *P* values in bold are statistically significant (<0.05).

*GVHD* graft-versus-host disease, *NRM* non-relapse mortality, *DFS* disease-free survival, *OS* overall survival, *ITS* immunosuppressive treatment, *CBT* cord blood transplantation, *HCT-CI* hematopoietic cell transplantation specific comorbidity index, *DRI* disease risk index, *TNC* total nucleated cell, *HLA* human leukocyte antigen, *HR* hazard ratio, *CI* confidence interval.

### Impact of discontinuation of IST on NRM and relapse

**Fig. 3:**
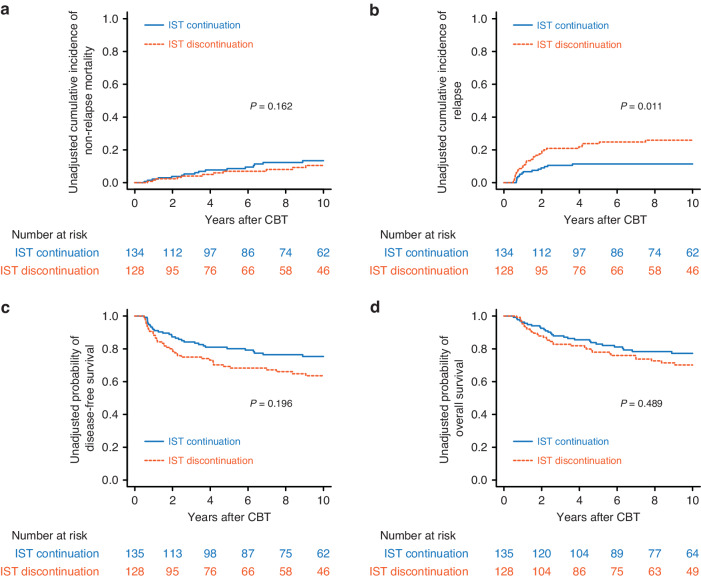
Outcomes after cord blood transplantation according to immunosuppressive treatment discontinuation. Unadjusted cumulative incidences of non-relapse mortality (**a**) and relapse (**b**), and probabilities of disease-free survival (DFS) (**c**) and overall survival (OS) (**d**) following single-unit CBT, according to IST discontinuation, plotted with a conditional landmark analysis at 180 days after CBT.

In the conditional landmark analysis at 180 days, the cumulative incidence of NRM at 5 years was 8.6% (95% CI: 4.5–14.3%) for patients who continued IST and 6.9% (95% CI: 3.2–12.6%) for patients who discontinued IST (*P* = 0.162 by Gray’s test) (Fig. 3A). In the multivariate analysis, discontinuation of IST was not associated with NRM (HR: 0.49; 95% CI: 0.20–1.21; *P* = 0.122) (Table 3). The cumulative incidence of relapse at 5 years was 11.4% (95% CI: 6.7–17.5%) for patients who continued IST and 23.8% (95% CI: 16.6–31.8%) for patients who discontinued IST (*P* = 0.011 by Gray’s test) (Fig. 3B). However, discontinuation of IST was not associated with relapse in the multivariate analysis (HR: 1.46; 95% CI: 0.61–3.46; *P* = 0.388) (Table 3).

### Impact of discontinuation of IST on DFS and OS

In the conditional landmark analysis at 180 days, the probability of DFS at 5 years was 80.1% (95% CI: 72.2–86.0%) for patients who continued IST and 69.2% (95% CI: 60.1–76.7%) for patients who discontinued IST (*P* = 0.196 by log-rank test) (Fig. 3C). In the multivariate analysis, discontinuation of IST was not associated with treatment failure (1-DFS) (HR: 0.87; 95% CI: 0.47–1.61; *P* = 0.676) (Table 3). The probability of OS at 5 years was 82.9% (95% CI: 75.2–88.4%) for patients who continued IST and 78.0% (95% CI: 69.3–84.5%) for patients who discontinued IST (*P* = 0.489 by log-rank test) (Fig. 3D). In the multivariate analysis, discontinuation of IST was not associated with overall mortality (1-OS) (HR: 1.91; 95% CI: 0.95–3.82; *P* = 0.065) (Table 3).

### Failure of IST discontinuation

**Fig. 4:**
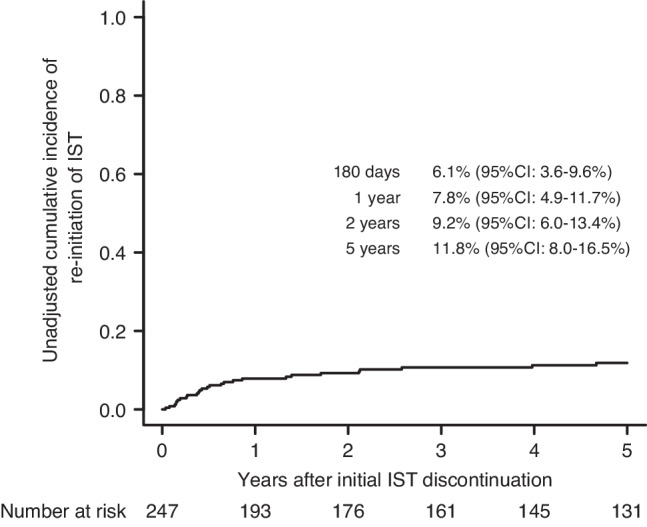
Incidence of re-initiation of immunosuppressive treatment (IST) following initial IST discontinuation. Unadjusted cumulative incidence of the re-initiation of IST following initial IST discontinuation after single-unit CBT.

Among the 247 patients who discontinued IST, 31 (12.6%) resumed IST, with a median time to re-initiation of IST at 777 days (range: 105–4670 days) after CBT. There was no significant difference in the median day of discontinuation of IST after CBT between patients who resumed IST and those who did not (165 days vs. 158 days, *P* = 0.618 by the Mann–Whitney *U* test). The median time to re-initiation of IST after initial discontinuation of IST was 231 days (range: 13–3473 days). The cumulative incidence of re-initiation of IST was 6.1% (95% CI: 3.6–9.6%) at 180 days, 7.8% (95% CI: 4.9–11.7%) at 1 year, 9.2% (95% CI: 6.0–13.4%) at 2 years, and 11.8% (95% CI: 8.0–16.5%) at 5 years after initial discontinuation of IST (Fig. 4). The main causes of the re-initiation of IST were the development of chronic GVHD (*n* = 24), cryptogenic organizing pneumonia or bronchiolitis obliterans organizing pneumonia (*n* = 4), and pneumonia caused by infections (*n* = 3). In the multivariate analysis using the Fine and Gray proportional hazards model, age ≥45 years was significantly associated with an increased incidence of re-initiation of IST (HR: 3.01; 95% CI: 1.22–7.37; *P* = 0.016), whereas the recent calendar year of CBT was significantly associated with a decreased incidence of re-initiation of IST (HR: 0.39; 95% CI: 0.17–0.92; *P* = 0.032) (Table 4). Of the 31 patients who resumed IST, 17 (54.8%) were able to discontinue it again, with a median duration of IST administration of 305 days (range: 11–2842 days), whereas 1 patient continued to receive IST. Among the remaining 13 patients, 10 died of NRM, and 3 experienced a hematological recurrence of the underlying hematological disease while receiving IST.

**Table 4:** Univariate and multivariate analysis of re-initiation of IST.

	Univariate analysis		Multivariate analysis	
	HR (95% CI)	*P*-value	HR (95% CI)	*P*-value
Requirement of steroid therapy				
No requirement of steroid	Reference		Reference	
Requirement of steroid	1.50 (0.72–3.14)	0.280	1.36 (0.59–3.14)	0.470
Age at CBT				
<45 years	Reference		Reference	
≥45 years	2.63 (1.23–5.60)	**0.012**	3.01 (1.22–7.37)	**0.016**
HCT-CI				
0–2	Reference		Reference	
≥3	2.17 (0.88–5.38)	0.092	1.60 (0.55–4.68)	0.380
Disease type				
Myeloid	Reference		Reference	
Other than myeloid	1.06 (0.49–2.29)	0.870	1.28 (0.55–2.93)	0.560
Refined DRI				
Low/Intermediate	Reference		Reference	
High/Very high	1.17 (0.57–2.38)	0.660	1.10 (0.49–2.46)	0.810
Cord blood TNC				
<2.5 × 10^7^/kg	Reference		Reference	
≥2.5 × 10^7^/kg	0.78 (0.38–1.58)	0.500	0.87 (0.42–1.77)	0.700
HLA disparities				
0, 1	Reference		Reference	
2	1.28 (0.59–2.76)	0.530	1.38 (0.59–3.20)	0.450
Sex incompatibility				
Female donor to male recipient	Reference		Reference	
Others	0.94 (0.43–2.05)	0.890	0.97 (0.44–2.14)	0.950
ABO incompatibility				
Match, minor mismatch	Reference		Reference	
Major, bidirectional mismatch	1.58 (0.78–3.17)	0.200	1.62 (0.74–3.55)	0.220
Year of CBT				
1998–2009	Reference		Reference	
2010–2023	0.77 (0.38–1.54)	0.470	0.39 (0.17–0.92)	**0.032**

The *P* values in bold are statistically significant (<0.05).

*CBT* cord blood transplantation, *HCT-CI* hematopoietic cell transplantation specific comorbidity index, *DRI* disease risk index, *TNC* total nucleated cell, *HLA* human leukocyte antigen, *HR* hazard ratio, *CI* confidence interval.

## Discussion

The timing of IST discontinuation may depend not only on the presence of active GVHD but also on stem cell source and genetic relationship with donor. Previous studies have shown that HLA-mismatched unrelated donors and the use of peripheral blood stem cells (PBSC) were associated with lower rates of IST discontinuation [1, 3, 38]. Despite limited data on IST management after CBT, several studies, including our previous study have shown a significantly higher rate of IST discontinuation in patients who underwent CBT compared to those who received conventional adult donor HCT [2, 25, 26]. Our data also showed that the incidence of IST discontinuation was 79.3% at 5 years post-CBT, and the requirement for steroid therapy and early periods of CBT (1998–2009) were risk factors for IST discontinuation failure. Although the cause of the requirement for steroid therapy could mainly be due to the treatment for severe GVHD, or toxicity of CSP, the failure of IST discontinuation during the early periods of CBT may indicate a notable shift in IST discontinuation practice among treating physicians at our institute [39]. Thus, our findings suggest that the discontinuation of IST is a frequent occurrence among adult patients who underwent single-unit CBT.

Several studies have evaluated the effect of the timing of IST discontinuation on subsequent chronic GVHD in allogeneic HCT from adult donors [9–11]. Mengarelli et al. showed that discontinuation of IST over 12 months led to an increased risk of subsequent extensive chronic GVHD in allogeneic PBSC transplantation from matched sibling donors [11], whereas Kansu et al. did not find a difference in subsequent chronic GVHD between a 6-month and 24-month course of CSP prophylaxis in allogeneic bone marrow transplantation from matched sibling donors or alternative donors [10]. In the context of CBT, our study demonstrated that discontinuation did not affect the subsequent extensive chronic GVHD. Moreover, 12.6% of patients resumed IST mainly due to developing chronic GVHD after initially discontinuing IST. This rate is lower than the previously reported data for adult donors, where 37.1% of patients resumed IST [3]. This difference might be partly due to the lower incidence of chronic GVHD in CBT [16–19, 21, 25, 26]. Therefore, our results suggest that the use of cord blood may promote higher immune tolerance without requiring prolonged IST.

Early dose reduction or discontinuation of IST could be an attractive strategy to augment the GVT effects, particularly for high-risk hematopoietic malignancies, as suggested by single-arm studies [4–7]. Moreover, the induction of GVL effects through the rapid tapering of IST has proven beneficial for treating post-transplant relapse for certain hematological malignancies [40–42]. However, the majority of previous comparative studies failed to confirm the efficacy of early dose reduction or discontinuation of IST in reducing post-transplant relapse in allogeneic HCT from adult donors [3, 11]. In contrast, Inamoto et al. showed that the discontinuation of IST prevented relapse in patients without GVHD during the first 18 months after HCT [8]. In our analysis of CBT among patients who survived at least 180 days without relapse, discontinuation of IST was associated with an increased risk of relapse in the univariate analysis but did not maintain this association in the multivariate analysis. Moreover, discontinuation of IST was associated with a trend toward overall mortality in multivariate analysis. These might be partly due to the selection bias inherent in retrospective studies because IST was discontinued early in patients with a very high risk of disease relapse. Therefore, a prospective interventional trial should be conducted to clarify the role of early dose reduction or discontinuation of IST in reducing post-transplant relapse in CBT.

Our study had several limitations. First, its retrospective nature implies that variations in clinical practices among treating physicians and disease activity may have influenced the timing of reductions and discontinuations of IST and the study’s outcomes. Second, our data only included single-unit CBT because double-unit CBT was not approved for use outside of clinical trials during the study period in Japan. Several studies have shown that double-unit CBT mediated a higher incidence of severe GVHD than single-unit CBT [43, 44]. Moreover, calcineurin inhibitors and MMF, but not MTX, were commonly used for GVHD prophylaxis for CBT in US and European centers [45, 46]. Recently, the annual number of CBT is decreasing around the world, because rates of HLA-haploidentical HCT continue to expand owing to the application of posttransplant cyclophosphamide for GVHD prophylaxis [47]. Therefore, we should exercise caution when extrapolating our findings to double-unit CBT or Western countries. Nonetheless, the inclusion of a homogeneous population of adult patients undergoing single-unit CBT using CSP-based GVHD prophylaxis with a median follow-up period of 10 years is a strength of our study.

In summary, our data showed that successful discontinuation of IST is relatively common after single-unit CBT in adults. Among patients who survived at least 180 days without relapse, discontinuation of IST did not affect subsequent extensive chronic GVHD, relapse, or survival, suggesting that discontinuation of IST within this timeframe is both feasible and safe for adults undergoing single-unit CBT in a real-world setting. A prospective interventional trial should be conducted to clarify the role of early dose reduction or discontinuation of IST in reducing post-transplant relapse in CBT. Our data can provide insight into the feasibility and safety of the early discontinuation of systemic IST after single-unit CBT.

## Data Availability

Data may be available from the corresponding author upon reasonable request.
